# Development and evaluation of a voice dataset for voice therapy exercises: quantitative audio analysis and user feedback from a cross-sectional study

**DOI:** 10.3389/fdgth.2026.1835534

**Published:** 2026-09-11

**Authors:** Sabrina H. Schröder, Rica Schulze, Merle Schlender, Torge Jensen, Frank Schulze, Navid Tabriz, Andrea Henze, Michael Buschermöhle, Sebastian Fudickar, Dirk Weyhe, Verena Uslar

**Affiliations:** 1 University Clinic for Visceral Surgery, Carl von Ossietzky University Oldenburg, Oldenburg, Germany; 2 Institute of Medical Informatics, University of Luebeck, Lübeck, Germany; 3 HelferApp GmbH, Gommern, Germany; 4 Nutritional Physiology, Martin-Luther-University Halle-Wittenberg, Halle/S., Germany; 5 KIZMO GmbH, Klinisches Innovationszentrum für Medizintechnik Oldenburg, Oldenburg, Germany; 6 Section for Clinical Research IT (SKFIT), Institute of Medical Biometry and Statistics (IMBS), University of Luebeck, Lübeck, Germany; 7 Fraunhofer Research Institution for Individualized Medical Technology and Engineering (IMTE), Lübeck, Germany

**Keywords:** biofeedback, digital health, dysphonia, mobile application, open access dataset, speech and language pathology

## Abstract

**Purpose:**

This study aims to develop and validate a high-quality voice dataset for dysphonia therapy using a standardized, video-guided recording protocol comprising a range of therapeutic voice exercises. The dataset is designed to support the development of digital health applications that enable home-based voice training with real-time biofeedback, thereby addressing current challenges in speech-language pathology, including limited access to therapy and the need for scalable, patient-centered interventions.

**Methods:**

A voice dataset was collected from adult participants performing voice therapy exercises in German, recorded via smartphone and in-ear microphones under standardized conditions. In addition to acoustic data collection, participants evaluated the recording procedure and video-guided exercise instructions. Acoustic analyses of pitch (Hz), intensity (dB), and Cepstral Peak Prominence (CPP) were conducted using Praat, with data grouped by age and sex. Correlation analyses were performed to assess the consistency of recordings across participants and exercises.

**Results:**

The resulting dataset comprises recordings from 175 adults, totaling approximately 6.6 GB of audio data, with a subset made available as open access. Participants reported high overall acceptance of the recording procedure and instructional videos, while also identifying areas for improvement, particularly regarding pacing and clarity. Acoustic and correlation analyses demonstrated exercise-specific variations in CPP, pitch and intensity, as well as systematic differences across age and gender groups, indicating both variability and structured consistency within the dataset.

**Conclusion:**

The dataset represents a novel resource for digital voice therapy, particularly due to its focus on structured therapeutic exercises in German. It provides a robust foundation for the development and evaluation of digital health applications aimed at enhancing self-managed voice training and supporting therapy processes beyond clinical settings.

## 1 Introduction

Dysphonia is characterized by a temporary or chronic impairment of voice functionality, accompanied by changes in voice quality. This condition has a substantial impact on an individual's capacity for effective communication and results in a diminished quality of life. The causes may be organic (e.g., complications following thyroid surgery) or functional (e.g., prolonged voice strain among professional speakers) (1). The associated costs of primary healthcare for dysphonia amount to about 5 billion USD annually worldwide (2).

Speech and Language Pathologists (SLPs) typically treat dysphonia in outpatient settings. To facilitate faster therapeutic outcomes, patients are regularly assigned tasks for supplementary self-training at home without the ability to confirm the correct exercise execution (1).

At the same time, demographic change is expected to increase the demand for speech and language therapy services. In particular, population ageing is likely to result in a higher prevalence of voice disorders, as the risk of dysphonia increases with age (3, 4). Simultaneously, an increasing shortage of SLPs may further limit access to speech and language therapy services (5). As a result, the gap between the supply of and demand for therapy will be further exacerbated, meaning that therapy cannot be provided in a timely manner—or at all. Patients would suffer from this undersupply and experience limitations (6) in their daily participation and quality of life. In this context, supporting effective self-training at home becomes increasingly important. Digital tools such as mobile applications could complement conventional therapy by guiding patients through exercises, supporting structured home practice, and potentially reducing the burden on SLPs while maintaining therapeutic support between sessions. Given this potential, further research in this field is needed to understand how digital therapeutic solutions can improve patient care in the future (5, 6).

Based on this, the app demonstrator OLA (Oldenburger Logopädie App—Oldenburg Speech Therapy App) was developed. The focus of OLA was to provide exercise videos for voice therapy, which can be individually activated for patients by their treating SLP. Both user groups, SLPs and patients, have evaluated the acceptance, usability, and user experience of OLA, with both expressing a willingness to use a digital assistance system like OLA in the future (7).

Building on this, the LAOLA^1^ project was initiated to further develop and enhance digital support for voice therapy. To guide this development, a needs analysis involving 29 SLPs and six patients was conducted. Participants provided recommendations regarding the design, structure, and accessibility of a voice therapy app (8).

Accordingly, LAOLA expands the existing concept of OLA by offering a broader range of exercises, a more modern video presentation, and separate instructional videos for individual exercises. In addition, both SLPs and patients expressed a need for objective voice feedback during home practice. Therefore, one of the key features planned for LAOLA is the provision of objective real-time feedback on voice production, accompanied by a performance overview to support self-monitoring.

To enable such feedback mechanisms, reference data describing typical acoustic characteristics during correctly performed exercises are required.

In particular, recordings from vocally healthy individuals can provide sucha baseline by capturing the expected acoustic patterns of the exercises.

Several speech datasets -Saarbrücken Voice Database (9), Massachusetts Eye and Ear Infirmary, AVID (Aalto Vocal Intensity Database), HEART database, and Arabic Voice Pathology (2, 9)—have been created for voice analysis and speech processing research. While these databases provide valuable resources, they differ substantially in terms of language coverage, participant characteristics, accessibility, and recording procedures. For example, the Saarbrücken Voice Database is the only database containing German speech but lacks SPL calibration tones, while the Massachusetts Eye and Ear Infirmary dataset includes participants only up to the age of 59 years. In addition, some databases do not provide open access.

Beyond this, existing voice databases mainly focus on a limited number of diagnostic tasks, such as tone-holding phonation, speak of automatisms and read sentences aloud (2, 9). As a result, the acoustic patterns that occur during structured therapeutic voice training are largely underrepresented in current datasets. Therefore, the present study aims to generate an initial voice dataset of voice therapy exercises recorded by predominantly vocally healthy participants with a wide age range among the participants.

In contrast to existing databases, the dataset includes a broad spectrum of voice therapy exercises performed by vocally healthy participants following standardized video instructions in the German language. To evaluate its suitability for digital voice therapy applications, the dataset is further examined using complementary qualitative and quantitative analyses. These analyses assess participants' experiences with the video-guided recording procedure as well as the consistency of the acoustic perceptual parameters obtained from the recorded exercises.

Thus, the study addresses the following research questions:

*Can a high-quality voice dataset of exercises for dysphonia therapy be generated using a standardized video-guided recording setup?*

1. *How do participants evaluate the recording procedure and the video-guided exercise instructions used during data collection?*
2. *How consistent are the acoustic perceptual parameters of the recorded voice data across exercises?*

While the findings directly support the further development of the LAOLA app demonstrator and similar digital voice therapy tools, the generated dataset and the accompanying analyses also provide broader value for voice therapy research. In contrast to many existing speech datasets that focus on isolated phonemes or words, the present dataset captures complete therapy exercises, offering a more realistic representation of therapeutic voice training and enabling future research on automated feedback in voice therapy. With this article, the corresponding voice dataset is made available via open-access^2^, contributing to the scientific investigation of voice analysis in digital dysphonia therapy.

## 2 Methods

The methodology of this study was designed to generate and evaluate a structured voice dataset of voice therapy exercises. To assess the quality and suitability of the generated dataset, the recordings were evaluated on two complementary levels. First, a qualitative evaluation examined how adult participants perceived the recording procedure and the video-guided exercise instructions used during data collection. This evaluation provides insights into the usability and acceptance of the recording setup as well as the clarity of the exercise guidance. Second, a quantitative evaluation was conducted by analyzing acoustic perceptual parameters derived from the recorded voice data. This analysis allows us to examine the acoustic characteristics of the exercises and to assess the consistency of the recordings across participants.

The following sections describe the study design, the selected voice therapy exercises, the recording setup, and the procedures used for qualitative and quantitative evaluation.

When preparing the manuscript, established reporting guidelines were considered to ensure transparency and reproducibility. In particular, the Template for Intervention Description and Replication (TIDieR) checklist (10)^3^ was used to describe the intervention and recording setup. The reporting of the study design was guided by the Transparent Reporting of Evaluations with Nonrandomized Designs (TREND) statement (11). For the qualitative component of the study, namely the participant survey, the Standards for Reporting Qualitative Research (SRQR) (12) were followed.

### 2.1 Study design

The study was approved by the medical ethics committee (2023–171) of the University of Oldenburg and of the University of Lübeck (2023-713), and all participants provided written consent. The study is registered in the German Register of Clinical Trials (DRKS00033225)^4^. Between June and October 2024, recruitment took place through flyers and personal outreach at the Pius Hospital in Oldenburg and in external testing settings in Lower Saxony. Recordings were conducted at these same locations. Following an information session and the signing of consent forms, sociodemographic data were collected. The test subjects and the research team speak German as their mother tongue. The first author (SHS) is herself an SLP and has played a key role in conducting the study.

As the primary aim of this study was to generate a voice dataset of voice therapy exercises performed by vocally healthy adults (18–35, 36–65, >66 years) to establish baseline acoustic characteristics, recruitment focused mainly on vocally healthy participants. The rating “vocal health” based on the participants' subjective self-assessment and the clinical assessment of the lead author, who is an SLP, and another SLP from the team. Both SLPs always agreed on the assessment. The assessment of the SLP is based on an unremarkable (respectively 0 points) GRBAS (Grade, Roughness, Breathiness, Asthenia, Strain)-scale. In addition, a small number of patients undergoing voice therapy and SLPs were included to obtain an initial impression of the feasibility of the recording procedure in a realistic therapeutic context. However, the acoustic analyses presented in this study focus on the recordings of vocally healthy participants. Exclusion criteria include minors, individuals with poor or no German language skills, and persons who, due to cognitive or physical limitations, are unable to cope with the duration and intensity of participation. In addition, professional singers were excluded, as the practice repertoire does not include any vocal exercises. Following the recommendations of the “Clinical and Laboratory Standards Institute”-guideline (13), we aimed to recruit at least 120 participants in order to enable a robust description of acoustic voice parameters and to establish normative acoustic baselines.

### 2.2 Selection of exercises

In the aforementioned needs analysis (8), participants were introduced to the LAOLA concept and asked which voice therapy exercises they considered particularly suitable for guided home-based practice using instructional videos. It was emphasized that SLPs using LAOLA can subsequently select different exercises tailored to the individual patient.

Consequently, exercises were selected that do not require additional materials, such as a therapy ball, and can be conducted while maintaining a relatively stable position, without jumping or moving around. The selection of exercises also took into account the available evidence. Consequently, recommendations from German voice therapy guidelines (14) and other sources (e.g., 1) within the field of voice therapy in Germany were considered. Furthermore, SLPs suggested during the needs analysis that singing exercises be deliberately omitted, as this constitutes a separate area of therapy and is not relevant for all patients (8). Therefore, LAOLA concentrates exclusively on special functional training. As the situational context (e.g., dealing with emotional reactions in certain conversational situations) and personal aspects (e.g., adopting vocal role models) remain part of present therapy, we will omit these aspects in this work.

The selection of exercises was primarily guided by therapeutic relevance and suitability for independent home practice rather than by technical considerations regarding automated feedback generation. As LAOLA is intended to provide feedback based on both acoustic and visual information, the exercise set includes not only voice-based exercises but also exercises focusing on posture, movement, and body tension that are commonly used in dysphonia therapy. As the present study focuses on the generation of an acoustic voice dataset, only exercises containing analyzable voice recordings were included in the quantitative analyses.Focusing on the therapeutic areas of breathing, posture/tone/movement, articulation, and phonation (1), subsequently fifteen exercise videos were included. These exercises are summarized as follows (Table 1).

**Table 1:** Overview of the selected exercises (phonation is relevant in the exercises marked in bold) *(own presentation).*

Type of the exercise	Description	Number of exercise-videos
Stretching exercises	Stretching the neck, upper body, and arms for the reduction of muscle tension	2
Activation of the diaphragm	Quick, targeted arm movements in rhythm	2
	**Diaphragm training Rhythmically adjusted phonation for the promotion of deep reflective breathing during the production of syllables**	
Fröschels' impact exercises	**Targeted voice strengthening (support of glottal closure) through coordinated mechanical arm movements**	3 *with different levels: sound/syllable, word, sentence*
Fröschels' chewing method	**Development of supraglottic space and normalization of vocal tract muscle tone through chewing movements with and without phonation**	2 *with different levels: sound/syllable, word*
Improvement of the mobility of the articulatory organs	**Velum exercises: Activation of the velum through the phonation of syllable chains combined with arm movements and the expansion of the chest for the improvement of resonance and articulation**	4
	Tongue motor skills: movements of the tongue tip, partly in diadochokinesis, e.g., “licking teeth”, for the promotion of mobility and strength, support for clear articulation and voice control	
Semi-Occluded Vocal Tract Exercises (SOVTE)	**SOVTE trains the interplay of the essential aspects of vocalization: breathing, voice production, and resonance formation. A steady driving pressure combined with a constant airflow velocity forms the basis for this and is achieved through exercises involving resistance against air (or water).**	2
	**Movements involving the lips or corners of the mouth, partly in diadochokinesis, e.g., “lip trills”.**	

During the recording of the training videos in a dedicated studio at the University of Oldenburg, feedback from the OLA study (7) was taken into account. Consequently, instructional and exercise videos were recorded separately. To create contrast against the light-marbled background and between the two types of videos, the model wore a red cardigan with a black (instruction) or white (exercise) T-shirt. Additionally, a green plant was placed in the background of the instructional videos. During the editing of the videos, the items (sounds, syllables, words, or sentences) to be imitated were captioned at the top center. To provide variety, a calm melody was played during exercises where phonation or speech was not required.

### 2.3 Recording setup

Due to the necessity of reliable and valid measurement of vocal expressions—both from a patient-oriented and socio-economic perspective—the objectivity, reproducibility, and practicality of acoustic analysis methods are of central importance for evidence-based clinical practice. Successful application requires a clear definition of which perceptual parameters are measured, how these relate to dysphonia, and what threshold values differentiate pathological from physiological voice performance. The ease of use of the measurement technology, the comprehensibility of the results, and the associated costs are also relevant (15). Correspondingly, we ensured that the voice recordings for subsequent acoustic analyses were conducted in an environment with minimal sound reflections and reduced reverberation, to minimize the influence of room acoustics (16).

In this context, several 15 m^2^ office rooms in different buildings were used as recording environments, with each participant being alone in the room during the recordings. The use of different locations reflects the intended everyday application of the LAOLA app demonstrator, in which users should be able to train their voice in various environments, provided that disruptive background noises, such as a playing radio, are avoided. At the same time, recording in different rooms introduces a certain degree of variability into the data, which can contribute to adapting the app to real-life settings.

During the testing, participants wore Bluetooth in-ear headphones with integrated microphones from the brand Poounur with default settings, thus, only capturing the participants' voices, not that of the training-video instructions. In addition, the in-ear headphones ensure a standardized and consistent microphone-to-mouth distance (16, 17) of 10 cm, which is recommended in voice research (16). At the same time, the recordings were conducted using readily available consumer electronics rather than specialized audio hardware in order to reflect realistic usage conditions. Consequently, the dataset captures voice recordings under conditions that are both standardized and representative of everyday use.

The participants were positioned in front of two adjacent tripods: Firstly, the iPhone 14 Pro recorded RGB (red, green, blue) and depth data of the test subjects with two front cameras and stored this locally on the iPhone together with the audio file. For this work, the voice dataset is of significance and is checked for quality. Secondly, the Microsoft Azure Kinect camera recorded detailed depth image data. This is important for the later video analysis, as the data was collected in the same setting. In a subsequent step within the LAOLA project, visual feedback (e.g., posture), should also be made possible. This will take place with the support of artificial intelligence, which is not yet relevant in this paper. However, the video data generated here from the participants is already very helpful for this purpose.

### 2.4 Prior to the recording

**Figure 1:**
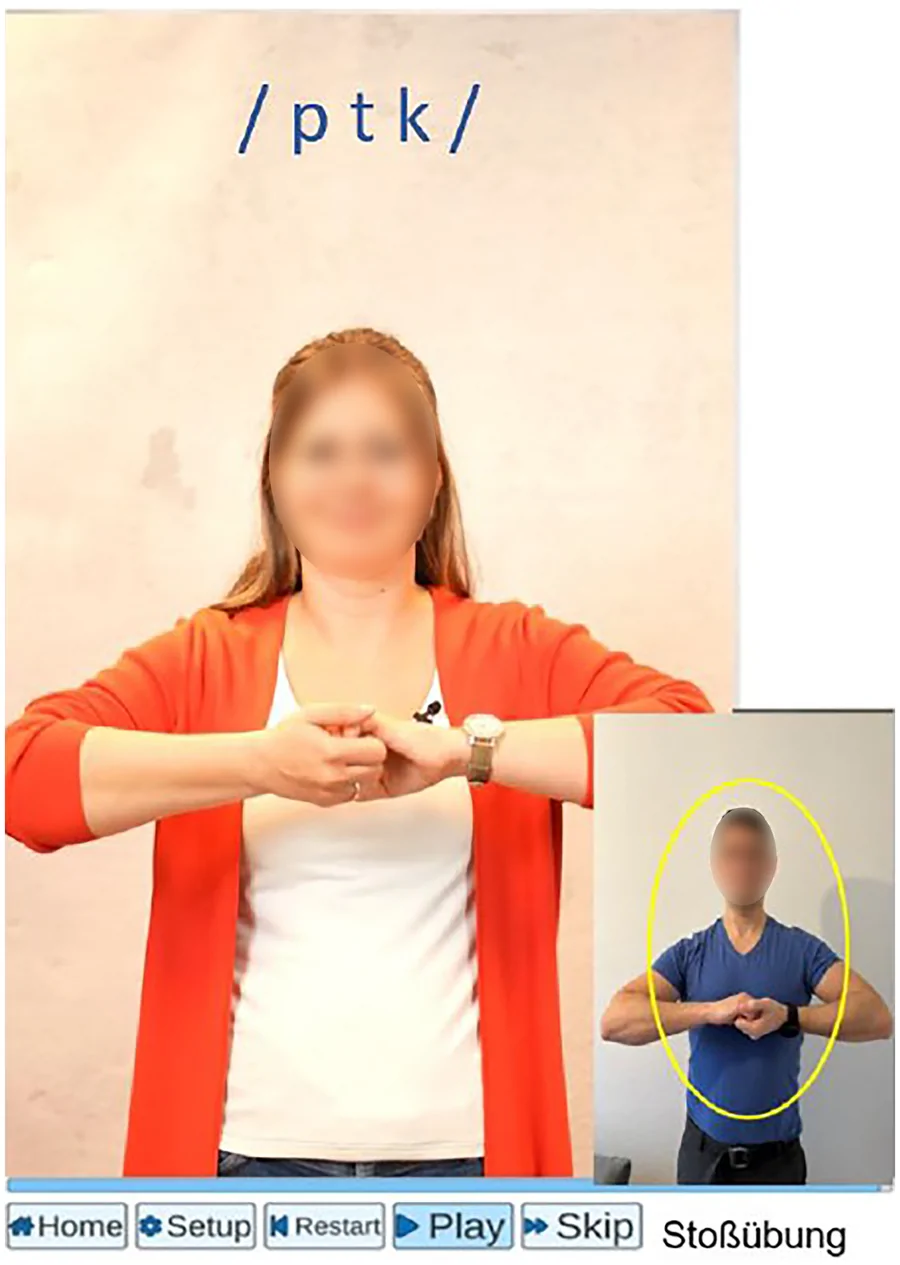
Screenshot of the app showing a video and the participant who sees himself in the mirror function (bottom right) during the Fröschels' impact exercises.

Participants were allowed to complete the recordings either standing or sitting, and the selected posture was documented accordingly. This decision was made to reflect the anticipated use of the LAOLA app in everyday therapeutic practice, where some patients may be unable or unwilling to perform exercises while standing (e.g., due to physical limitations or postoperative conditions). Additionally, even in in-person therapy, the decision to seat or stand is made individually per exercise for each patient, taking into account their capabilities and goals (1). Participants were positioned such that they were visible from the crown of the head to the navel in a frontal view. During the instructions, a small window on the bottom right of the iPhone screen allowed the participant to view themselves, providing the opportunity to correct their position using an overlay template (an oval circle) (Figure 1).

In order not to interrupt the exercises due to long upload times, all videos were subsequently uploaded to a secure data server of the LAOLA project.

The participants were initially reminded verbally and in writing to speak at a normal intensity (neither whispering nor shouting). Next, the informational video on physiological posture^5^ was shown to, so that everyone was reminded of the important posture for phonation in the same manner. Subsequently, the recording began with 14 instructional videos (average duration: 1.0 min) and 15 exercise videos (average duration: 1.5 min). The order of these 15 exercises (Table 1) was randomized across participants to reduce potential sequence effects associated with completing all exercises within a single recording session. Thereby we intended to reduce potential influences of general fatigue, habituation, or changes in attention over time. There was no possibility of repeating the exercises.

### 2.5 Qualitative evaluation of the recording setup

**Figure 2:**
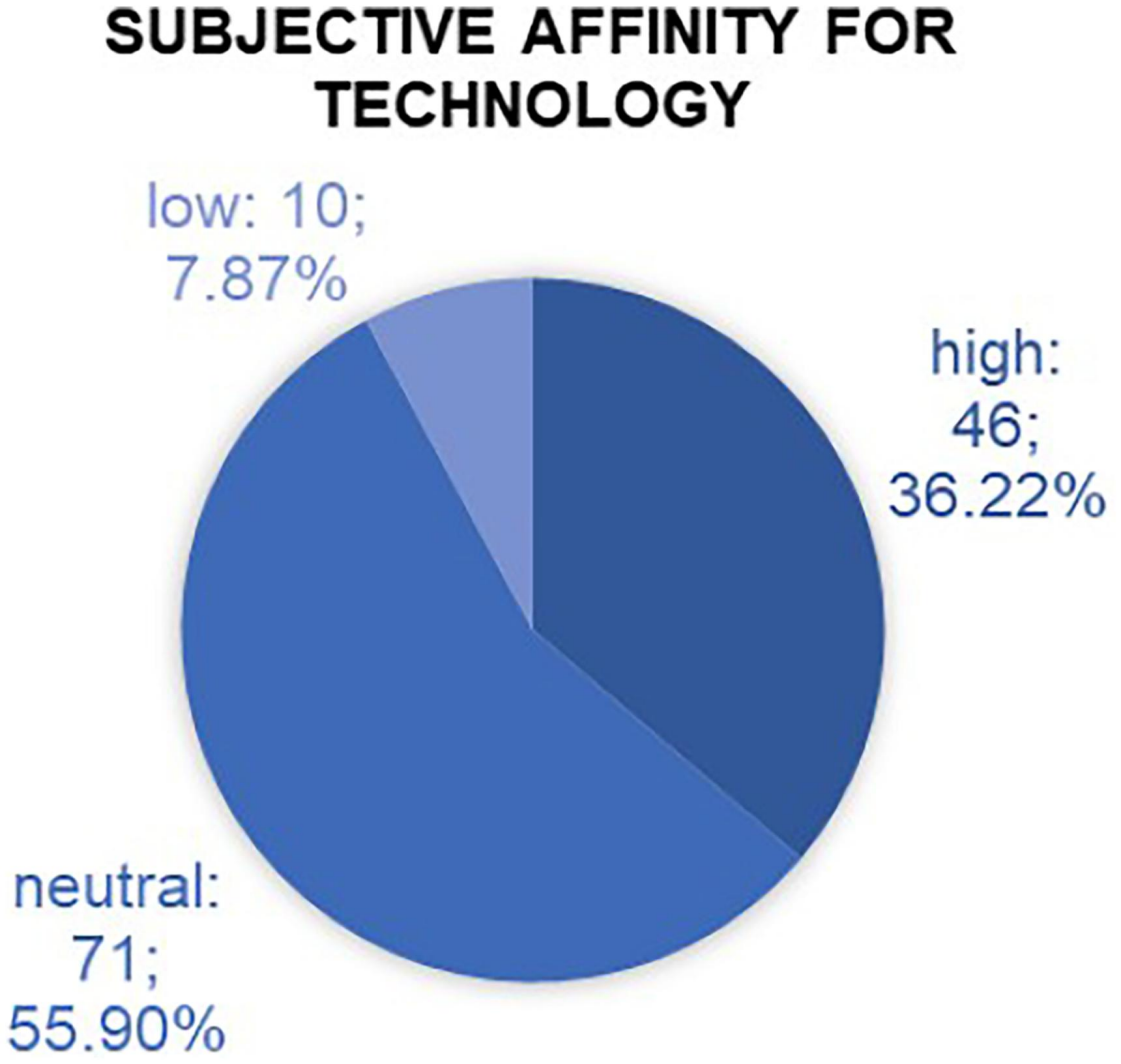
Subjective affinity for technology (total population).

Finally, participants were given a self-designed follow-up questionnaire for personal assessment of the videos and the test setting, which was analyzed descriptively. The questionnaire comprised 16 items with a four-point Likert scale (“yes, very” to “no, not at all”) without a midpoint. The items were chosen based on the framework of clinical reasoning that is used during voice therapy to promote self-reflection among patients (1). Finally, the participants evaluated the test situation and the videos in a brief informal feedback discussion. Furthermore, the participants provided a subjective assessment of their own affinity for technology (“good”, “neutral”, “poor”; Figure 2), and the researchers (SHS, RS) noted external features, such as piercings in the face or body weight, which could be relevant for later video analysis.

### 2.6 Quantitative acoustic analysis

Objective acoustic analysis is often used alongside subjective treatment assessment in clinical settings (16). Such analyses commonly include acoustic parameters such as pitch, intensity and Cepstral Peak Prominence (CPP), which reflect important characteristics of voice production and are frequently used in the assessment of voice disorders (1, 18, 19). As normative data derived from calibrated microphones in standardized environments cannot be transferred to the everyday use of the application, we calculated exercise-specific comparison values for our own values (CPP, pitch, and intensity) for the respective exercise within the application. This is because factors such as microphone characteristics, microphone positioning, recording distance, and environmental noise may substantially influence acoustic measurements (9, 15).

For the extraction and analysis of the acoustic parameters, Praat (20) was used. It facilitates comprehensive examinations of speech signals, including the analysis of quantitative measures like frequencies, formants, and pitches, and offers functions for editing audio recordings after the file has been uploaded to the program.

The following acoustic parameters pitch, intensity, and CPP (Table 2) were selected because they represent the primary acoustic dimensions targeted by the investigated therapeutic voice exercises, including velum exercises, Fröschels' chewing method, Fröschels' impact exercises, and diaphragm training/flow phonation. The aim of the acoustic analysis was to characterize exercise-related vocal behavior. Therefore, parameters reflecting modulation of pitch and intensity, as well as overall voice quality, were considered most relevant for the present study. The entire phonation in the files was used for the analysis. For comparison, the normative data of the European Laryngological Society (21), Hammer and Teufel-Dietrich (1), and Murton et al. (18) is included in Table 2.

**Table 2:** Acoustic parameters incl. definition and normative data *(own presentation oriented towards* 14, 18).

Parameter	Definition	Normative data
Pitch (mean)	The fundamental frequency is the vibration rate of the vocal folds, measured in hertz (Hz). It determines the pitch of the voice.	♂: 73–294 Hz ♀: 165–659 Hz
Intensity (Sound Pressure Level, SPL)	The intensity describes the sound pressure level of the voice, measured in decibels (dB). It depends on sub-glottic pressure regulation and articulation.	intensity *in conversations:* ca. 60–70 dB (A) *Whisper:* <50–55 dB (A) *Shout:* >80–90 dB (A) *Note:* In the field of spoken voice, a decrease and increase in intensity of up to 30 dB SPL should be possible.
Cepstral Peak Prominence	CPP captures overall periodicity of the voice; one of the best-validated acoustic predictors of dysphonia and also works with continuous speech.	Dysphonia with CPP <9

Dysphonic voice signals are characterized by an increase in irregularities of intensity and fundamental frequency. Fundamental frequency is the most robust measure even under suboptimal recording conditions, as it allows the data to be compared between different audio file formats, hardware and software devices. In contrast, the interpretation of intensity is more sensitive to recording conditions such as microphone characteristics and room acoustics. As a result, acoustic voice parameters are influenced by various technical and environmental factors, which complicates direct comparisons across studies or with reference values reported in the literature (15). Therefore, the subsequent analysis focuses on the parameters pitch, intensity and CPP to characterize the acoustic properties of the generated dataset and to allow a cautious contextualization of the results with respect to values reported in previous studies.

To analyze the acoustic data, the audio tracks were first extracted from the video recordings and converted into.wav format using FFmpeg. Then, the recordings were normalized using Audacity to standardize recording levels prior to further acoustic analysis. As the recordings were obtained using consumer-grade Bluetooth microphones employing automatic gain control (AGC), and because normalization alters the original amplitude characteristics of the signal, intensity measurements cannot be interpreted as calibrated sound pressure level (SPL) values. Consequently, intensity measures were considered relative acoustic descriptors only and were not used for direct comparison with normative SPL data.

Each audio recording was then analyzed using Praat (20). Intensity and pitch were determined for all recordings (minimum, maximum, mean, standard deviation). In addition, we included the median and interquartile range to better capture the data characteristics, especially in the presence of asymmetries or outliers. CPP was also calculated using Praat with the settings provided by Maryn and Weenink (19), except that we decided against removing silent passages from the sound file before the calculation. The reason for this is that we will eventually need to process the signals in real time to provide real-time feedback to users, and real-time processing with speech pause removal is not currently feasible.

As voice characteristics vary with age, sex, and the presence or absence of voice disorders (1), participants with dysphonia were first excluded from the acoustic analysis in order to establish a baseline for vocally healthy individuals. The remaining participants were then grouped according to age and sex. Age was divided into three groups (18–35, 36–65, over 66 years), and sex into three categories (male, female, other). To increase the quality of the dataset, recordings with unusually low and high mean values for intensity and pitch were reviewed manually. They could indicate technical issues, incomplete recordings, or recordings containing little analyzable speech material. Recordings with mean intensity values below 45 dB received additional scrutiny, as recommendations for ambient noise levels during speech therapy assessments are typically reported in the range of 45–50 dB SPL (15, 16). For pitch, the study was guided by the normative data from Schneider & Bigenzahn (16): 73–294 Hz for males and 165–659 Hz for females (18). These values were used solely as a pragmatic point of orientation for identifying potentially unusual recordings. We found that some participants had started too late, or experienced technical issues related to microphone settings. These recordings were manually excluded from further consideration.

However, no conclusions regarding the actual ambient noise levels of the recording environments can be drawn from this threshold. Ambient noise was neither measured nor controlled during data collection, as the study aimed to approximate realistic conditions for future home-based use of the LAOLA application rather than standardized laboratory conditions.

We conducted a supplementary analysis to examine the consistency of the temporal structure of exercise execution across participants. The aim was not to assess acoustic similarity between recordings, but rather to investigate whether participants followed the video-guided exercises with comparable timing and speech-pause patterns. Such consistency may be relevant for future automated analyses and feedback systems, as highly standardized temporal structures can facilitate the comparison and interpretation of recordings. This was done by randomly selecting twelve videos per exercise from each of the eight acoustic based exercises (stratification by age group and sex), and then comparing them using cross-correlation. The cross-correlations of the whole-time signals were calculated using a custom Matlab script that automatically read in the audio tracks of the twelve selected videos. Amplitudes of all sound files were then set to either 0 or 1 according to a threshold of 0.01. The threshold was chosen to suppress background noise and account for the noise floor of the recordings. This binarization allowed the analysis to focus solely on the presence or absence of speech events, as the cross-correlation was intended to compare the temporal structure of the signals rather than their absolute amplitude values. With these files we calculated the cross-correlation for all possible combinations of signals using the xcorr function for intensity. This resulted in 66 cross-correlation values per exercise, from which the maximum value and lag were determined. The mean and standard deviation of the 66 maximum values per exercise were calculated and graphically represented. The lags at the maximum values of the cross-correlation were checked for outliers.

## 3 Results

After the demographic data of all participants has been presented, their subjective feedback to the exercises and recording setup will follow, along with the presentation of the acoustic dataset. The majority (74%) agreed to make their audio data openly available. In this article, we present both, the entire audio dataset of the study as well as the open access subdataset. In a subsequent paper, the open access video data will then be presented and analyzed. This cannot be included in this work, as not all individuals who checked open access for audio recordings also checked open access for video data.

### 3.1 Demographic and statistical data

In this study, 175 recordings were made in total, which corresponds to approximately 53 h (≈6.6 GB) of audio data and 400 GB of data in total. Table 3 gives an overview of the participants' sex and age distribution of all participants and the subgroup that agreed to provide their audio recordings via open access.

**Table 3:** Overview of the sex and age of all subjects (Complete population vs. Open access for audio file) *(own presentation).*

	Complete population (175; 100%)	Open access for audio file (129; 74%)
Sex	♂: 45; 26%	♂: 29; 30%
	♀: 119; 69%	♀: 69; 70%
	other: 0; 0%	other: 0; 0%
Age	18–35 years: 80; 46%	18–35 years: 49; 50%
	36–65 years: 65; 37%	36–65 years: 39; 40%
	> 66 years: 21; 12%	> 66 years: 10; 10%

In terms of ethnicity, the complete cohort is homogeneously Caucasian with only 3 Arabic, 1 Latin American, and 1 Asian ancestry. In addition, five participants were physically impaired (sitting in a wheelchair or with restricted arm movement due to illness). As two other people were generally not in good health in^6^, they stopped the exercises early. The exercises they completed could still be used.

Participants' self-reported affinity for technology (Figure 2) revealed that 7.87% indicated a low affinity, 55.90% were neutral, and 36.22% reported a high affinity.

It was also asked which professional group the participants belong to. Many different professional groups were represented (see Appendix in Table A). A substantial proportion of participants came from the “Health, Social Services, Education, Training” category (21%). Professionals who rely on their voice, such as teachers and educators, fall within this category. SLPs are additionally listed within this group, accounting for 23%.

### 3.2 Feedback from the subjects to the exercises and recording setup

The feedback presented here from the participants refers to the results of the questionnaire (Table 4) and the brief informal feedback discussion. It supports the qualitative evaluation of the audio dataset and also offers insight into participants' expectations of a suitable recording environment and the design of app-based voice therapy training.

**Table 4:** Quantitative feedback from the subjects (the most common answer is marked in bold) *(own presentation).*

Question	Yes, very much.	Yes.	No.	No, not at all.
The **distance to the smartphone** was appropriate: I could see everything important.	**66** **(66%)**	31 (31%)	4 (4%)	0 (0%)
The **pace of the instructions** was reasonable.	47 (46%)	**48** **(47%)**	7 (7%)	0 (0%)
The **pace of the exercises** was appropriate.	24 (24%)	**58** **(57%)**	20 (19%)	0 (0%)
The exercises were **easy to understand**.	45 (44%)	**54** **(52%)**	4 (4%)	0 (0%)
I was **able to concentrate** well during the execution.	41 (41%)	**45** **(45%)**	14 (14%)	0 (0%)
The **duration of the individual exercises** was reasonable.	46 (44%)	**51** **(49%)**	7 (7%)	0 (0%)
I am **satisfied with my performance** of the exercises.	9 (9%)	**64** **(63%)**	26 (26%)	2 (2%)
I would have liked **help from an expert** during the exercises.	1 (1%)	12 (12%)	**57** **(56%)**	31 (31%)
I would have liked **feedback on the correctness** of the exercises.	7 (7%)	**44** **(45%)**	40 (41%)	7 (7%)
I would have liked **praise and motivation** during the exercises.	4 (4%)	20 (20%)	**47** **(47%)**	29 (29%)
Towards the end, **I felt more relaxed.**	13 (13%)	**46** **(47%)**	33 (34%)	6 (6%)
I had **fun** doing the exercises.	24 (37%)	**30** **(45%)**	12 (18%)	
I had **experience with such exercises** before.	25 (25%)	22 (22%)	23 (23%)	**30** **(30%)**
I had previous **experience with video and audio recordings** of myself.	19 (19%)	29 (29%)	**30** **(30%)**	22 (22%)
In everyday life, I often **need help using my smartphone**.	0 (0%)	5 (5%)	26 (26%)	**70** **(69%)**
I **like to use my smartphone**.	47 (46%)	**48** **(46%)**	7 (7%)	1 (1%)

The video instructions on physiological posture were, in general, considered helpful. They felt thereby reminded to continue paying attention to their posture.

For most respondents, the pacing of instructional videos (47%, with 46% finding it highly appropriate) and exercise videos (57%, with 24% highly appropriate) was suitable. However, for 19%, the exercise pacing was too fast. Participants recommended adding a distinct start cue, such as an audible countdown from three, as the initial part of exercises was often missed due to uncertainty about when they began. Exercise duration was appropriate for 49% (44% very appropriate), though several participants suggested shorter instruction videos with one example per exercise. The intended visual differentiation between instruction and exercise videos, marked by the instructor's variation of T-shirt color, was appreciated but insufficient. A color-changing border could enhance this effect further in their opinion.

Exercises were easy to understand for 52% of respondents (44% found them very easy), and 45% of respondents reported good concentration (41% very good) during recording, though some felt nervous. Feedback also indicated that the items to be repeated should appear earlier and stay visible longer in exercise videos, especially for *Fröschels' impact exercises at word level* and *diaphragm training*.

Respondents indicated that they would prefer to use their own breathing rhythm at their own pace without a video model rather than working in parallel with the model during breathing exercises. In some cases, it was difficult for them to speak simultaneously with the model. Therefore, requests were made to give more time to repeat speech exercises. In addition, the subjects would like to see a function in the next version that would allow them to repeat an exercise.

Some SLPs reported that the diaphragm training instructions could be misinterpreted and should be revised to reduce the risk of incorrect execution.

Exercises such as *SOVTE* and *Fröschels' chewing method* were unfamiliar to some participants, who were inexperienced with voice therapy or speech training. Overall, the training was often described as more strenuous than expected, including the *Fröschels' impact exercises* and *the diaphragm training*.

Some participants reported discomfort when using the in-ear headphones, and the headphones occasionally interfered with the execution of specific exercises. The distance to the smartphone was perceived as very appropriate (66%).

Several participants reported that the overall recording session was lengthy. Since all exercises were performed consecutively during data collection, potential fatigue or concentration effects cannot be completely ruled out. In the intended application, however, exercises are typically completed in shorter sessions of approximately ten minutes.

The participants' experience with self-video and audio recording was evenly split, with 48% experienced and 52% inexperienced. While most participants were voice-healthy, 49% expressed a desire for feedback on exercise accuracy or a mirror function for self-reflection, aligning with LAOLA's biofeedback concept. During exercises, 56% of respondents felt no need for expert support (31% none at all), and 47% did not seek encouragement or motivation.

By the end of testing, 47% felt more relaxed performing the exercises, 45% enjoyed them (with 37% greatly enjoying them), and 63% were satisfied with their exercise performance. Most participants enjoyed using their smartphones (46% very much, 46% yes) and required no assistance operating them (69%).

### 3.3 Acoustic dataset analysis

In the analysis of the acoustic data, we focus on the data of healthy voices (a total of 143 datasets, 125 datasets with open access). The acoustic dataset analysis of this study includes the identification of outliers, the intensity, pitch, and CPP as calculated via Praat (20), as well as the representation of the cross-correlation. Here is the link for the “LAOLA—voice therapy recording audio dataset 1”: https://doi.org/10.5281/zenodo.16614289.

Based on the aforementioned normative values for pitch and intensity (16, 18), data from our dataset were displayed that deviated from the norm. We manually removed actual outliers after reviewing the files. Dropouts usually occurred because participants either only participated after a certain time or not at all. Most outliers were found in Velum exercise 1 and the exercises of Fröschels' chewing method. Ultimately, 32 outliers, distributed across all exercises, were removed.

**Figure 3:**
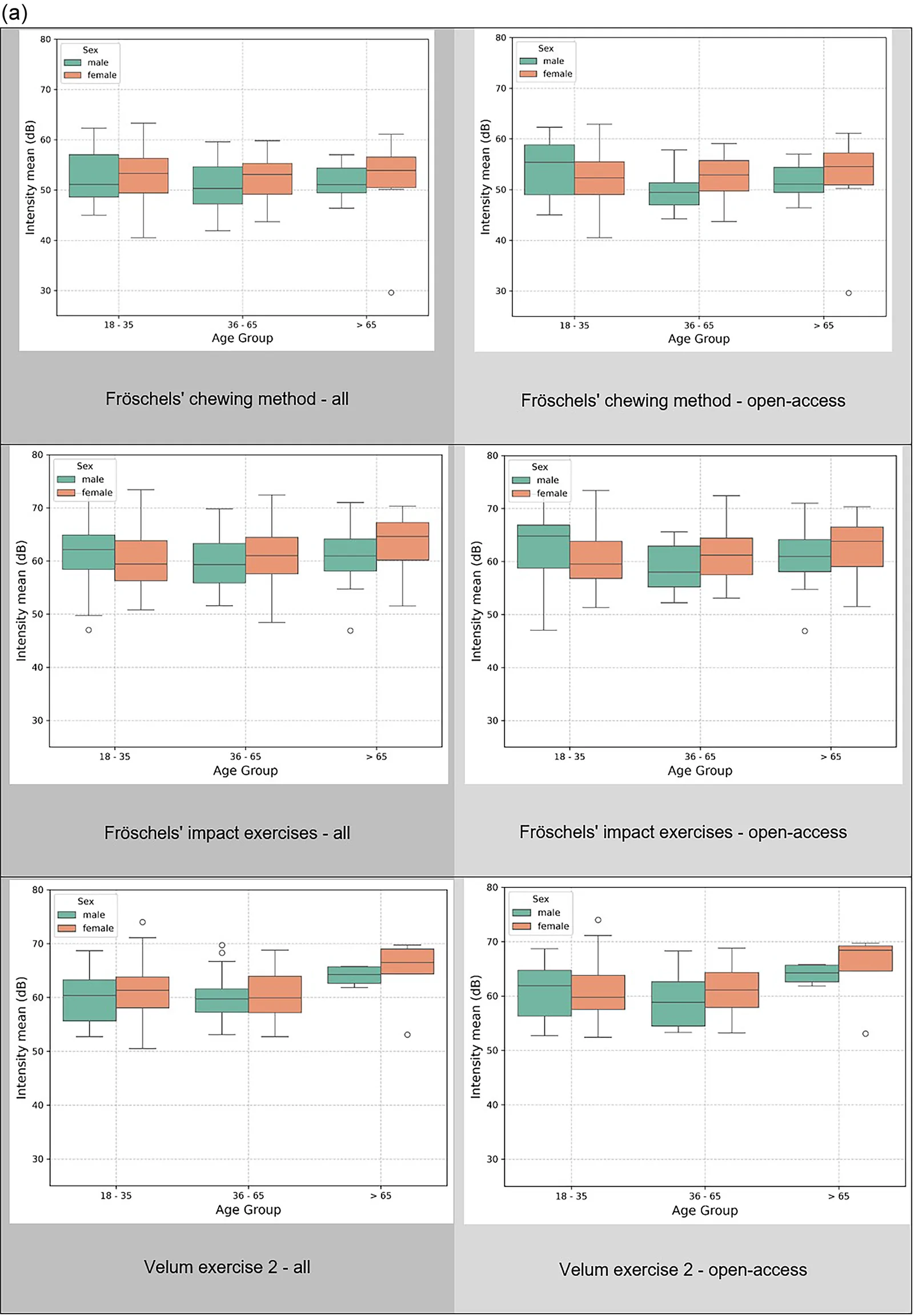
**(a)** Parameter “Intensity” for exercises, where the open-access dataset differs from the complete dataset *(own presentation)*. **(b)** Parameter „intensity“ in different exercises, when the parameters per exercise are almost identical *(own presentation).*

**Figure 4:**
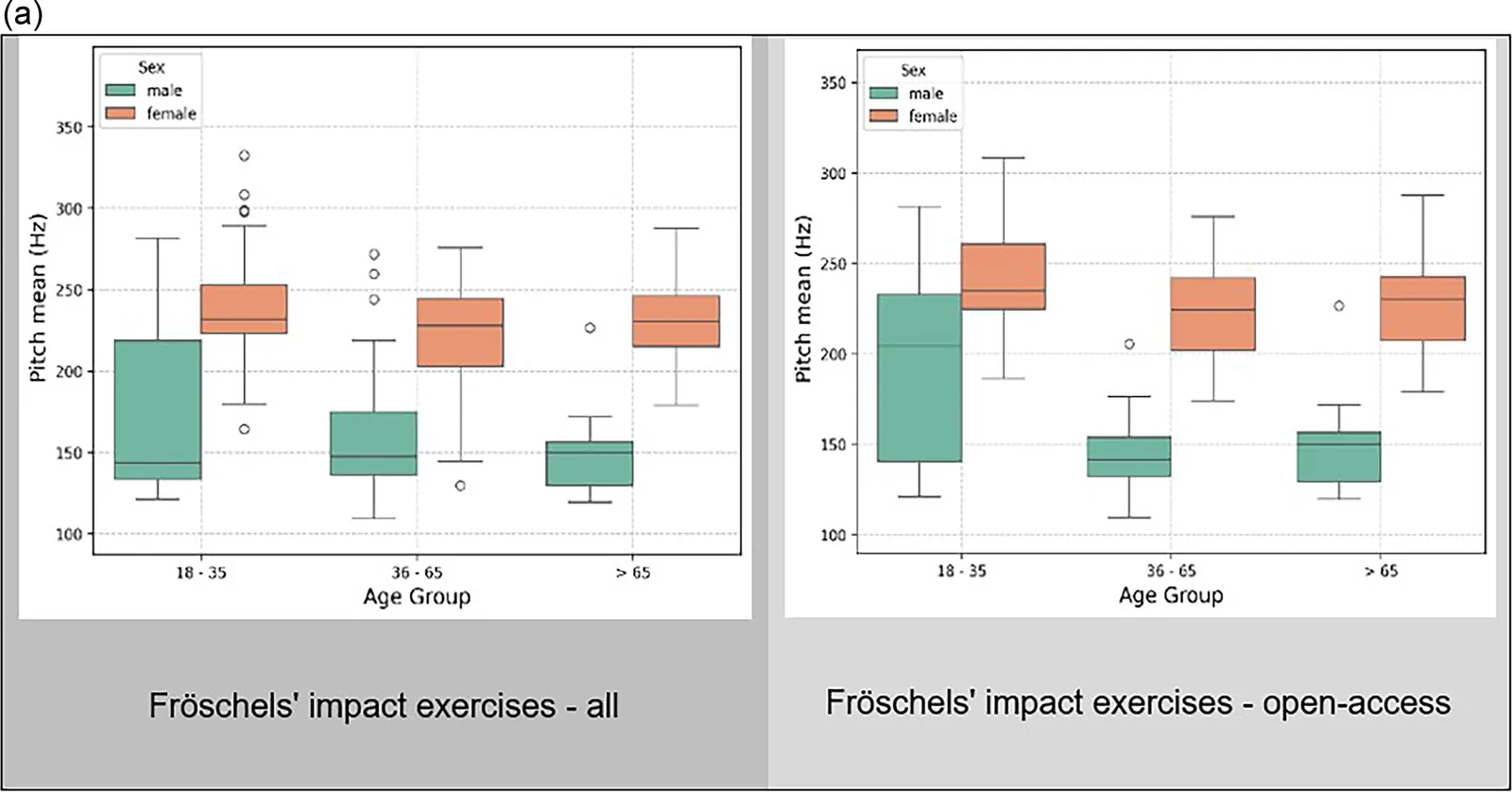
**(a)** Parameter “pitch” in exercises, when the parameters per exercise are almost identical *(own presentation)*. (**b**) Parameter “pitch” for exercises, where the open-access dataset differs from the complete dataset *(own presentation).*

The following figures show the corresponding results for both the general cohort and the subgroup that agreed to open access publication. The parameters “Intensity (in dB)” (Figures 3a, b) and “Pitch (in Hz)” (Figures 4a, b) are shown in box plots for each exercise (*Fröschels' chewing method, Fröschels' impact exercises, diaphragm training, velum exercises 1–3*). We decided to present the results exercise-wise since we expected different intensityand pitch in each exercise, due to exercise-specific characteristics. The three levels (sound/syllable, word, sentence) of *Fröschels' chewing method* and *impact exercises* were summarized. To improve clarity and avoid unnecessary complexity in the presentation of results, the open access and non-open access groups are shown separately only when statistically significant differences were found. Otherwise, results are reported jointly under the category “all”.

**Figure:**
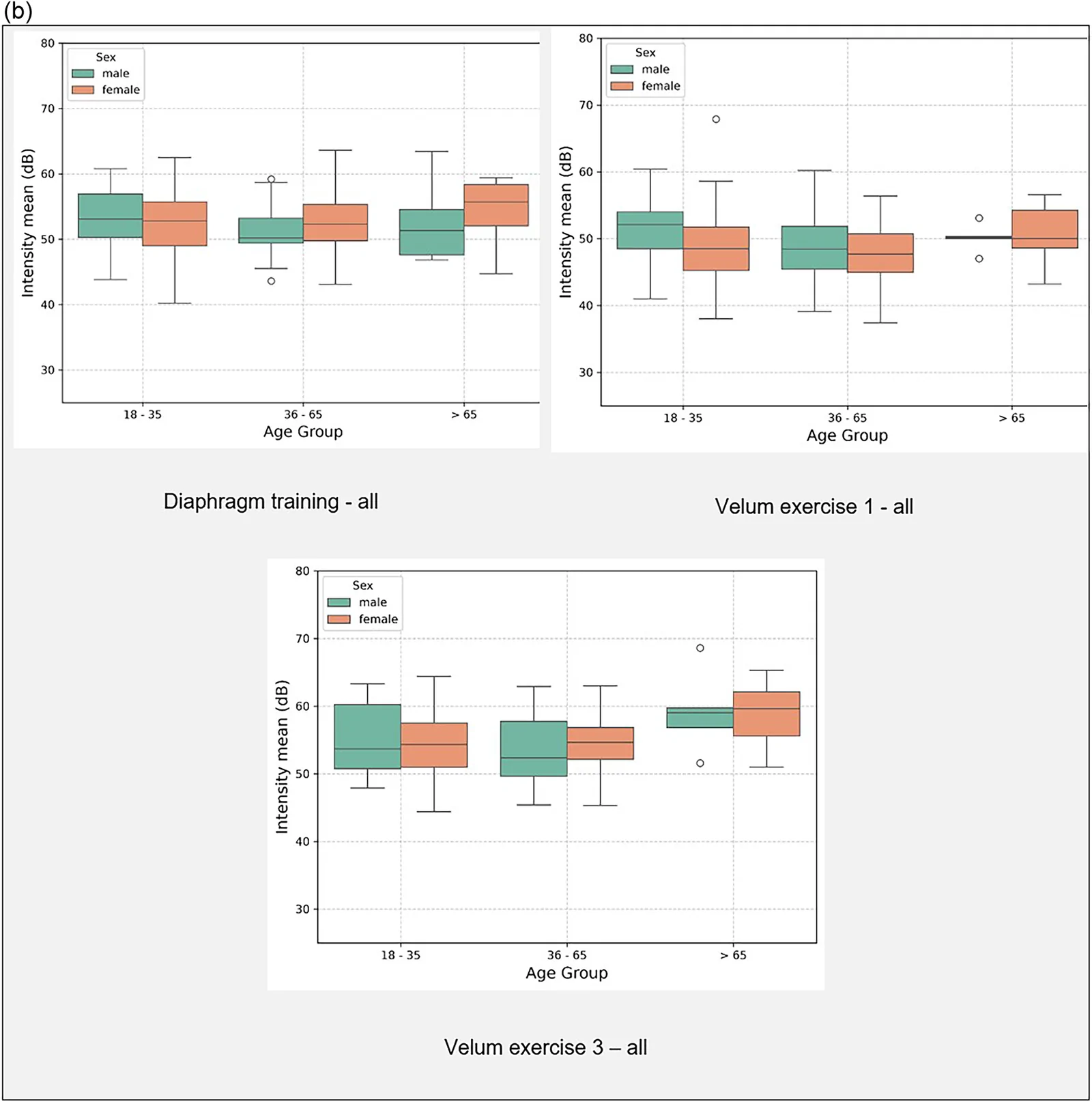


**Figure:**
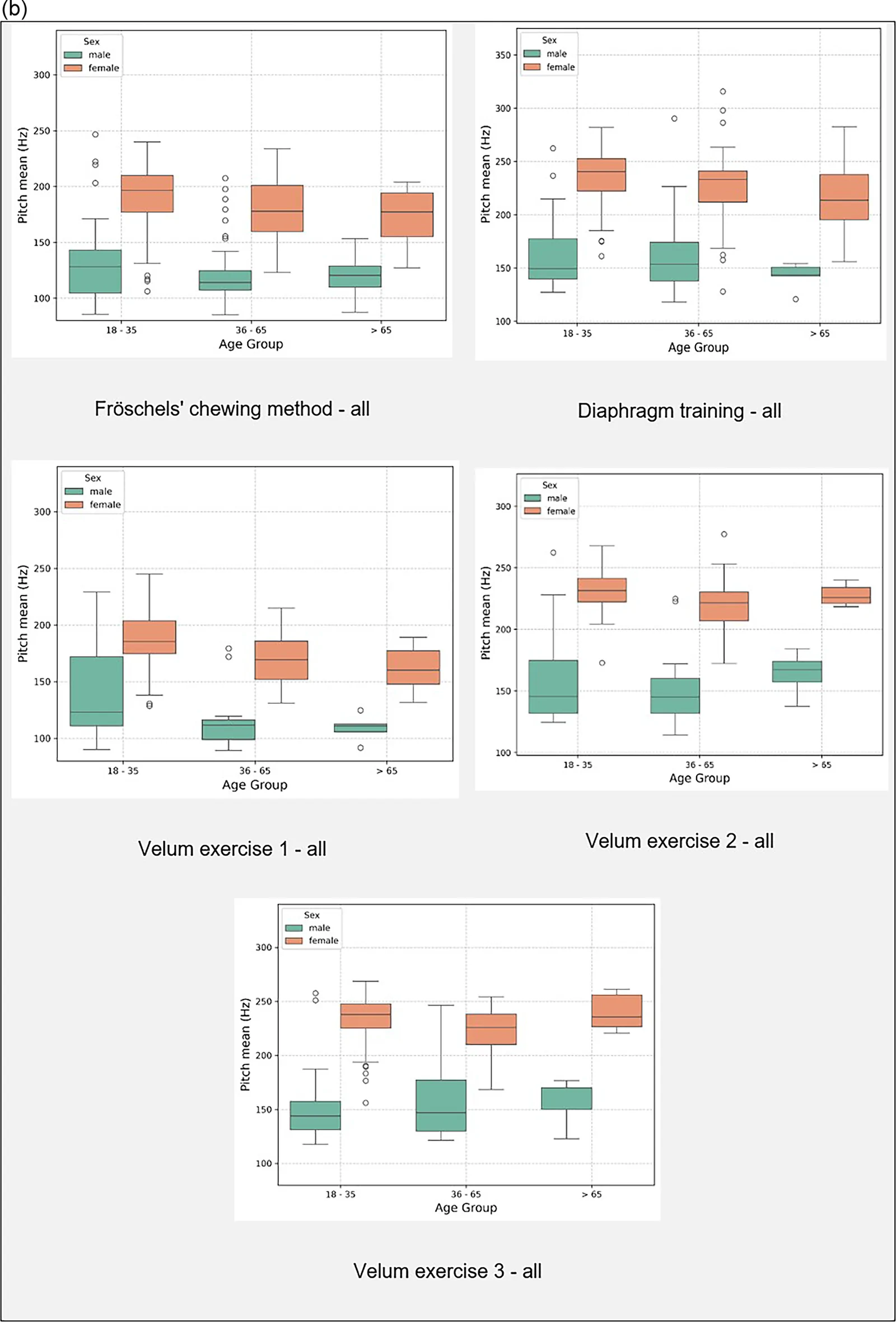


Overall, age and sex differences are more pronounced for pitch than intensity. The results of the cohort confirm that women have slightly lower intensity values than men—this corresponds to the normative data (1, 16).

The dispersion tends to be greater in the *velum exercises*, as different requirements were set for each exercise (multiple repetition of the syllable/ga/ on one breath and in one pitch vs. repetition of similar syllables, such as/haha/, with the last part being particularly emphasized vs. multiple repetition of the syllable/haha/ on one breath with movement of the arms and modulation of the prosody).

Figures 4a, b show the pitch (in Hz) during the execution of *Fröschels' impact exercises* and reveal sex differences for the full dataset: male participants consistently show much lower median pitch values across all age groups—approximately 140 Hz (18–35), 120 Hz (36–65), and slightly above 130 Hz (>65). In contrast, female participants display relatively stable median values between 200 and 220 Hz.

The open access subset differs in pitch dispersion, particularly among younger males (18–35), who show a noticeably wider range. In contrast, the female data appears more homogeneous, with fewer outliers. Median pitch values remain stable and largely consistent with the full dataset.

Overall, both visualizations confirm sex-specific pitch differences and an age-related decline in male pitch during midlife, followed by a slight increase in older age. Female pitch remains largely unaffected by age. The reduced variability in the open access data, especially among women, may be due to the smaller sample size of the dataset.

In terms of pitch, the boxplots for the *diaphragm training, Fröschels' chewing method* and *impact exercise* are very similar to each other—especially regarding the mean and spread within the age and sex groups (Figure 4b). In pitch, we found a clear difference between women and men for all exercises, as expected.. In men there are isolated lower values in the >65 group, but without a clear systematic shift across all exercises. The velum exercises 1–3 show a slightly larger range within the age groups in women (Figure 4b).

For logistical reasons, recordings were carried out in several rooms of equal size. A comparison of the recording environments revealed no differences in the distribution, dispersion, or occurrence of outliers for either pitch or intensity across the performed exercises, indicating that the different recording settings did not systematically influence the acoustic measurements.

The mean pitch values obtained from the LAOLA dataset (♂: 138.5 Hz, ♀: 209.5 Hz) were within the range of published normative data (♂: 73–294 Hz, ♀: 165–659 Hz). As the recorded intensity values did not represent calibrated sound pressure levels (SPL), no comparison with normative intensity data was performed.

**Figure 5:**
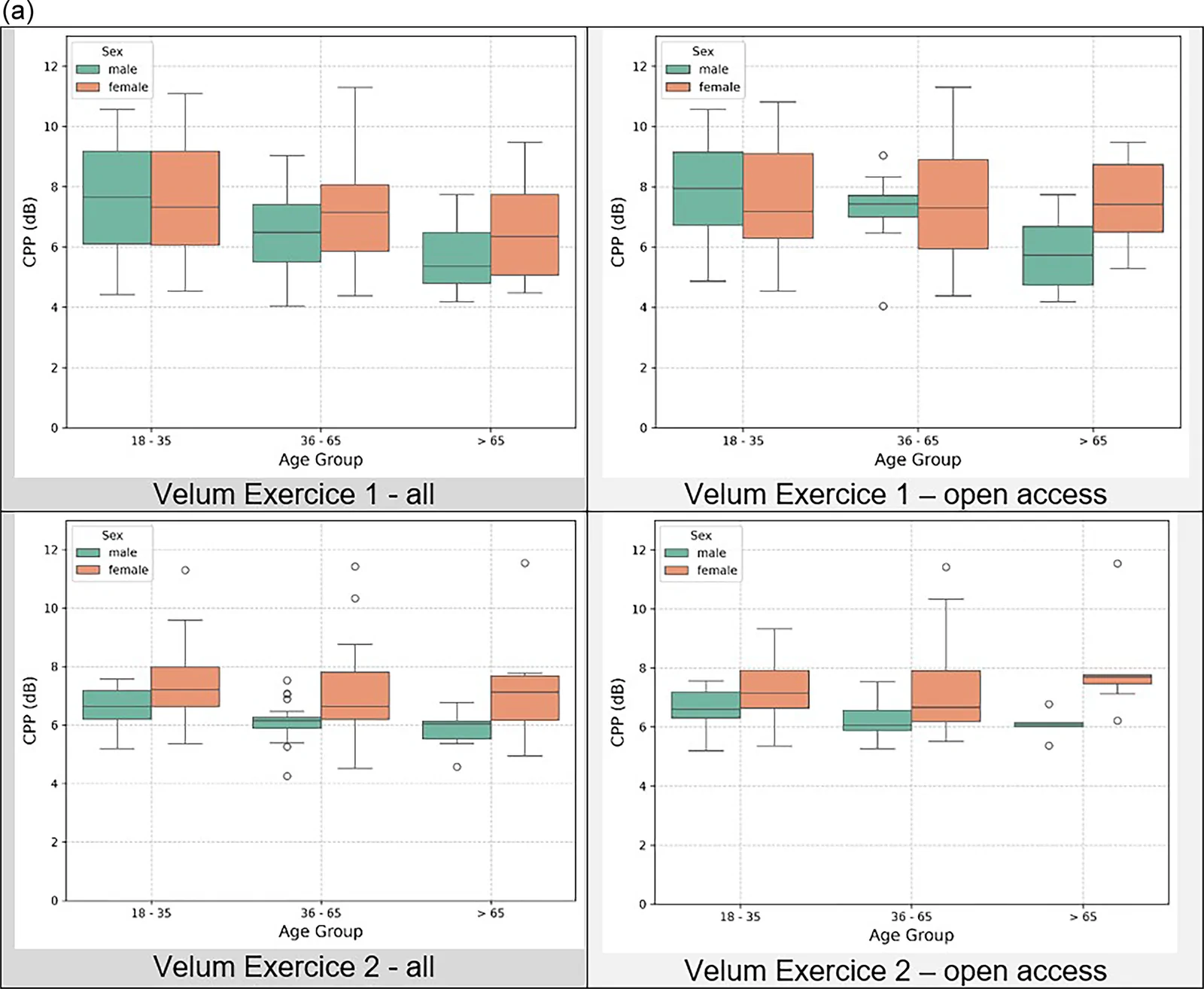
**(a)** Parameter “CPP” for exercises, where the open-access dataset differs from the complete dataset (own presentation). (**b**): Parameter “CPP” for exercises, where the open-access dataset differs from the complete dataset (own presentation).

The CPP analysis revealed differences between exercises as well as between age groups and sex (Figures 5a, b). Overall, median CPP values ranged between approximately 4 and 8 dB. Among the investigated exercises, Fröschels' chewing method and Velum Exercise 1 showed the highest CPP values, with median values generally between 6 and 8 dB. In contrast, diaphragm training and Velum Exercise 3 exhibited consistently lower CPP values, with medians of approximately 4–5 dB and comparatively small interquartile ranges. Fröschels' impact exercises and Velum Exercise 2 showed intermediate CPP values. Across most exercises, female participants demonstrated slightly higher median CPP values than male participants. Differences between age groups were generally small. However, Velum Exercise 3 showed greater variability in participants older than 65 years, whereas Velum Exercise 2 exhibited several high-value outliers across all age groups. In addition, female participants consistently showed higher median CPP values than male participants in Velum Exercise 2 across all age groups. Diaphragm training exhibited the smallest interquartile ranges of all investigated exercises, indicating a comparatively homogeneous distribution of CPP values among participants. In the open-access subset, CPP distributions remained comparable to those of the complete dataset, although younger male participants showed a somewhat wider dispersion than in the full cohort. Overall, the median values and distribution patterns of the open-access subset closely resembled those of the complete dataset across all investigated exercises.

**Figure:**
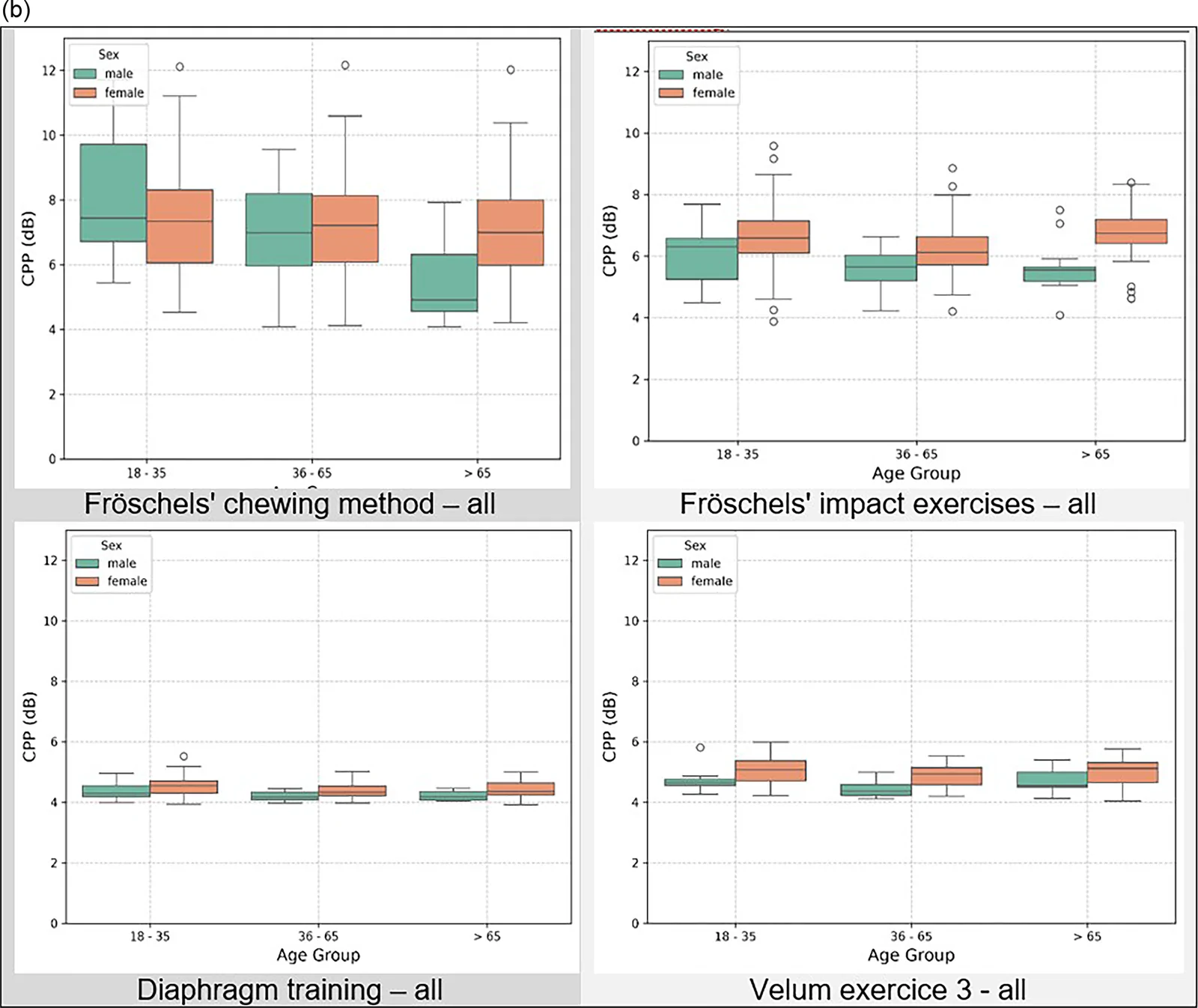


**Figure 6:**
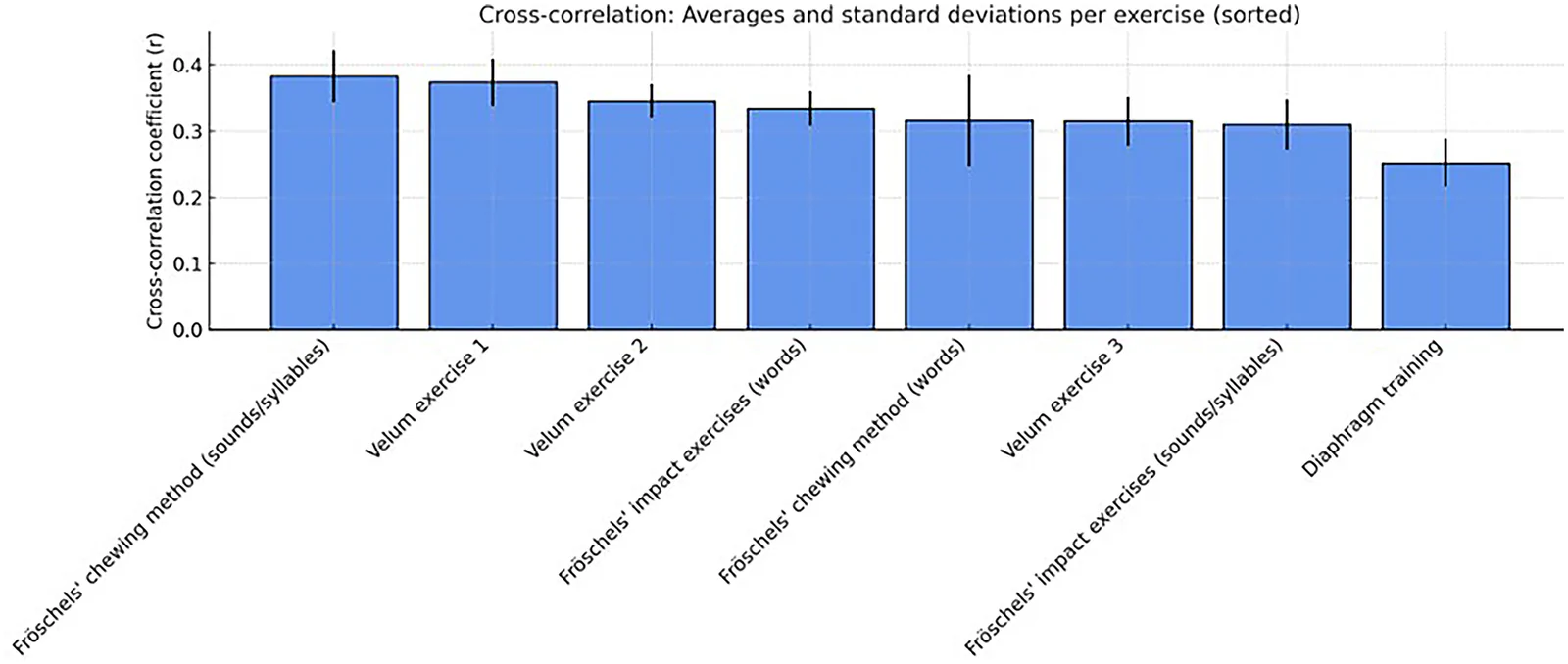
Bar chart with exercises (mean values and standard deviation) sorted in descending order according to cross-correlation—from the most strongly to the least strongly correlated exercise.

The acoustic data were also analyzed using cross-correlation. The bar chart (Figure 6) presents the mean cross-correlation values for each exercise together with their standard deviations (error bars). Higher cross-correlation values indicate greater similarity in the temporal structure of speech activity across participants, whereas larger standard deviations indicate greater variability between recordings. Because the analysis was based on binarized signals indicating the presence or absence of speech activity, the resulting cross-correlation values primarily reflect similarities in the temporal structure of exercise execution rather than acoustic characteristics such as pitch, intensity, voice quality, or performance accuracy.

- The exercises Fröschels' chewing method (sounds/syllables) (M = 0.383) and Velum exercise 1 (M = 0.374) showed the highest cross-correlation values. This indicates a comparatively high similarity in the temporal structure of speech activity across participants during these exercises.
- The exercises Velum exercise 2 (M = 0.346), Fröschels' impact exercises (words) (M = 0.334), Fröschels' chewing method (words) (M = 0.316), Velum exercise 3 (M = 0.315), and Fröschels' impact exercises (sounds/syllables) (M = 0.310) showed moderate cross-correlation values, which could indicate a moderate degree of similarity in the temporal structure of speech activity across participants. Fröschels' chewing method (words) exhibited the largest standard deviation, which could suggest greater variability in temporal execution patterns between participants.
- The diaphragm exercise (M = 0.252) showed the lowest cross-correlation values, which could indicate the greatest variability in the temporal structure of speech activity across participants.

The standard deviation shows how strongly the cross-correlation values scatter between the test subjects:

- Velum exercise 2 (SD = 0.025) and Fröschels' impact exercises (words) (SD = 0.026) showed the lowest variability, indicating relatively consistent temporal similarity patterns across participant pairs. In contrast, Fröschels' chewing method (words) (SD = 0.069) exhibited the highest variability, suggesting greater differences in the temporal structure of speech activity between participants.

Analysis of the lags at which the maximum values occurred showed that differences in the onset of the sound were all smaller than 1 s.

## 4 Discussion

### 4.1 Discussion of the feedback regarding study design and video training

Overall, participant feedback suggests that the recording setting was well accepted and suitable for collecting the audio data. The instructional material and general setup were evaluated positively, indicating that the study conditions were largely appropriate for obtaining recordings of good quality. For a more explicit description of people with healthy voices and those with voice disorders, it is recommended that in future studies the subjects be examined by an ENT specialist (e.g., using a laryngeal assessment) to assess their voice function.

The questionnaire responses showed no clear floor or ceiling effects and were broadly consistent across participants, suggesting that the feedback captured differentiated but comparable evaluations of the study setting. Therefore, the answers showed a high level of a high level of consistency among the participants. The inclusion of participants with different backgrounds, including patients, SLPs, and laypersons, added further value by combining lived experience with professional perspectives, although future studies would benefit from a larger and more diverse patient sample to strengthen generalizability. In addition, participants generally reported positive attitudes toward smartphone use in everyday life and rarely required assistance in handling their devices. This is in line with previous findings reported in the literature (22) and suggests that app-based voice therapy training may be acceptable and feasible for many users. At the same time, the feedback provided several practical indications for improving the user experience in future data collections. In particular, participants noted that shorter recording sessions about 20 min would be preferable, which corresponds with the average training time for home training. Furthermore, the use of in-ear headphones was not always comfortable. Five individuals with bilateral hearing aids had to remove them for testing; an aspect not initially accounted for in the study design.

In addition, visual support during the exercises, such as the mirror function implemented in the LAOLA app demonstrator (Figure 1), appeared to be important for monitoring posture and positioning during the recording. Participants confirmed its necessity for tracking posture. For head-focused exercises, a secondary template would also be beneficial. A further relevant observation was that some participants were uncertain about the start of individual exercises and occasionally missed the beginning of a task. This point is also important for interpreting the dataset, as it may help explain some of the outliers observed in the audio data analysis when participants started too late or did not perform the exercises as intended. Furthermore, participant feedback indicated that uploads should be made after recording to avoid unnecessary waiting time. Taken together, the feedback not only supports the overall suitability of the recording setting, but also highlights aspects that could be refined in future study designs and app-based voice therapy tools.

### 4.2 Discussion of voice data characteristics of the voice dataset

The acoustic analysis was deliberately restricted to voice-healthy participants, as the aim of this study was to establish a baseline for this novel type of audio data, namely standardized voice therapy exercises recorded under semi-controlled, ecologically valid conditions. This provides a reference framework for typical acoustic patterns in these tasks before extending the analysis to pathological voices. It should be noted that no instrumental or laryngeal examination was performed during the recruitment of participants. Incorporating such examinations is recommended for greater transparency and scientific rigor in future studies. As all subjects in this study speak German as their native language, linguistic and phonetic confounding variables are minimized, which increases the reliability and validity of the results. This enables more precise measurements of typical voice characteristics and standardized materials. However, generalizability to other languages is limited. Nevertheless, the scoping review by Kent et al. (23) suggests that their findings are very similar to the normative pitch data for English-speaking individuals. Moreover, a more balanced ethnic distribution could have enriched the dataset.

Furthermore, while the predominance of female participants reflects the demographics of voice therapy patients (1), it restricts conclusions about male and gender-diverse populations. Future research should therefore include speakers of different languages and a more balanced gender distribution to enhance cross-linguistic and cross-population applicability.

In order to evaluate therapy outcomes in an international context, systematic and valid assessment methods based on standardized protocols are essential (16, 17, 23). However, quality assurance in voice diagnostics proves to be more complex than in other areas of medicine. On the one hand, it is argued that the multidimensional nature of voice and speech makes it difficult to define universally applicable criteria and standards. Furthermore, additional costs—such as those for professional microphones—can pose a practical barrier for many SLPs (16). On the other hand, several established protocols and recommendations for instrumental and perceptual voice assessment are available (e.g., (17). Moreover, it is recommended that research targeting the standardization of acoustic data collection integrate audio- and video-documented procedures (like in this project), thereby improving the reproducibility and consistency of data collection across clinical and research contexts (23).

Our findings help to characterize the generated data set, which we are making publicly available. This offers future researchers the advantage that methodological aspects (e.g., microphone-mouth distance, comparability with normative data, differences from other datasets) remain transparent, enabling a better understanding of the conditions and limitations under which the data can be applied. Beyond the dataset itself, the results also contribute to the further development of the LAOLA project. In particular, the generated recordings provide an initial reference basis for future work on automated feedback mechanisms intended to support patients during home-based voice training. In addition, the qualitative findings provide valuable insights into the usability, comprehensibility, and practical implementation of the LAOLA concept. Participant feedback identified several aspects of the video design, exercise instructions, and recording procedure that can be refined in future iterations of the application.

Grillo et al. (24) investigated the use of various recording devices—such as iPhones and head-mounted microphones—alongside freely available software tools like Praat (20) for analyzing voice performance in male and female participants. In their study of daily voice monitoring (e.g., sustained phonation of/a/ and recitation of a standard sentence), they found that both, iPhones and head-mounted microphones in combination with Praat (20) enabled consistent acoustic measurements. To reduce variability, the authors recommend maintaining consistency in device type and application, as well as using a fixed mouth-to-microphone distance for each individual. These findings support the methodological approach used in the LAOLA app demonstrator, which likewise emphasizes consistent recording settings across sessions.

Additionally, Kodali et al. (9) reported similar methodological challenges regarding microphone distance and calibration in the context of SPL estimation. In their study, SPL values were shown to decrease systematically by approximately 6 dB with each doubling of the mouth-to-microphone distance, in line with the inverse distance law. To address this confounding factor, the authors employed both reference and test microphones, combined with calibration tones, and demonstrated that machine-learning models could partially compensate for distance-related variability. Their findings underscore that uncalibrated or smartphone-based recordings without standardized microphone placement inevitably introduce discrepancies in SPL measurements, highlighting the critical need for methodological control or correction when interpreting intensity data (9).

As demonstrated in the international literature on the subject (9, 15–17, 24), it is becoming evident that the distance between the microphone and the subject has a considerable influence on the measurement of sound pressure level (SPL) in this particular context.

Therefore, for our further development, we will focus on maintaining a uniform microphone distance for users and implementing a calibration function in the app. It will be clearer for users to receive feedback on the trends of vocal performance (e.g., “louder/quieter than before”) rather than absolute dB values. In the performance overview or documentation, we will transparently inform them that deviations from normative data may also be attributable to technical factors.

The incorporation of machine learning, such as to automatically perform classification or regression tasks using the acoustic speech signal (9), is an exciting aspect. This aspect could be incorporated into a future expansion of the LAOLA app demonstrator for acoustic feedback.

In light of these challenges, our study was carefully designed based on a transparent and literature-based framework. Aligned with the recommendations by Schneider and Bigenzahn (16) the study employed a Praat-based system for voice analysis, offering an objective and reproducible method. In doing so, the study contributes to the ongoing development of practicable and standardized acoustic assessment methods in the field of voice therapy. From both the participants' and researchers' perspectives, our recording setup proved effective even under realistic conditions, such as variations in video recording distance, room acoustics, and minimal background noise. The fact that the recordings in different rooms do not present significant acoustic differences regarding CPP, pitch and intensity, suggests that the dataset is robust under varying but acoustically comparable recording conditions. Beyond its relevance for dataset quality, this finding also supports the feasibility of home-based voice training applications, including app-based approaches such as LAOLA, when similar acoustic conditions can be maintained.Given the use of non-calibrated smartphone microphones, automatic gain control, and amplitude normalization, the recorded intensity values cannot be interpreted as absolute sound pressure levels (SPL). Therefore, comparisons with normative SPL data are not meaningful. Instead, the intensity data are reported descriptively, indicating a range of approximately 13 dB across the different voice therapy exercises. This variation is particularly evident in articulatory exercises, where lower vocal intensity is anticipated due to the controlled and clinical nature of the setting. Long speaking pauses, as they sometimes occur in our exercises, were not included in the calculation of mean intensity values. Furthermore, recording conditions, including ambient noise and device-specific sensitivity characteristics, may have contributed to systematic differences in the measured intensity values. Consequently, the LAOLA data should not be directly compared with laboratory-derived normative data obtained under standardized, noise-insulated conditions, as the recordings do not allow conclusions regarding absolute SPL levels. In the diaphragm training and Fröschels' impact exercises for instance, higher mean values are noticeable, which could indicate the conscious use of breath support and muscle activation. This further supports the rationale for establishing exercise-specific normative data based on our own recording protocol. Although recording conditions can demonstrably influence acoustic measurements, the findings of Awan et al. (25) indicate that modern smartphones, particularly recent iPhone models, can be used to obtain high-quality speech recordings suitable for informative acoustic analyses. Furthermore, Awan et al. (25) demonstrated that clinically relevant acoustic data can be derived from smartphone recordings when standardized recording protocols and appropriate quality-control procedures are implemented (25).

Our analysis showed that a large proportion of the pitch values we obtained were generally within the normative range although individual outliers were observed, especially in men aged 65 and above and during specific exercises such as *Velum Exercise 1*. These deviations may reflect age-related phonatory changes (1) or the acoustic demands of particular tasks, such as velum exercises or Fröschels' chewing method, which involve atypical articulation patterns and pitch modulation. Additionally, exercises conducted at the sentence level exhibited broader pitch variation compared to those performed at the sound or syllable level, likely due to prosodic variation in connected speech. Men consistently show lower values, which can be explained phoniatrically and physiologically by the lower pitch of the speaking voice. In women, a slight increase in pitch can be observed in the oldest group (>65 years)—consistent with previous studies describing age-related changes in voice pitch (1). In men, on the other hand, there are isolated lower values in the >65 group, but without a clear systematic shift across all exercises. The velum exercises 1–3 show a slightly larger range within the age groups in women, possibly due to greater inter-individual performance or vocal involvement (Figure 4b). Velum exercise 3 in particular shows more variance in older participants—possibly an indication of increasing muscular or coordinative challenges in old age (Figure 3b). The open access subset differs in pitch dispersion, particularly among younger males (18–35), who show a noticeably wider range. This may indicate increased heterogeneity within that subgroup. In contrast, the female data appears more homogeneous, with fewer outliers. Median pitch values remain stable and largely consistent with the full dataset, suggesting comparable voice performance across conditions.

This study focuses on the future documentation of voice performance within the therapeutic process, rather than on diagnostic voice assessments *per se*. Based on our results, an intra-individual comparison between the sessions per exercise may be enabled later in the app demonstrator. Through acoustic analysis of the recordings of healthy test subjects, we have established a baseline for different exercises. These exercises involve predefined phonation tasks—such as the articulation of sounds, syllables, words, or sentences in a specified manner (e.g., with particular prosody)—and differ substantially from spontaneous or read speech. Consequently, the measured acoustic parameters were collected and analyzed separately for each exercise type. Thus, we have developed a tool that assists in creating standardized recordings; the individuals perform the same actions at the same time. Our cross-correlation analysis showed that the participants all began speaking or making the corresponding vocal sounds at very similar times and then spoke at a similar rhythm. The diaphragm exercise (M = 0.252) showed the lowest cross-correlation values, which could indicate the greatest variability in the temporal structure of speech activity across participants. This suggests that participants followed the temporal pattern of this exercise less consistently than in the other exercises. However, the present analysis does not allow conclusions regarding the specific causes of this variability.

Close examination of outliers in the first raw audio data revealed particular difficulties with *Velum exercise 2* and the *Fröschels' chewing method*, which some participants did not complete.This could indicate a need to provide clearer instructions in the next stages of development. From a methodological standpoint, the observed discrepancies between measured values and standard norms should not be interpreted as erroneous but rather as a reflection of the exercise-specific and therapeutic context in which the recordings were made.

The CPP results indicate that cepstral characteristics depend not only on participant characteristics but also on the specific voice exercise performed. Across almost all exercises, female participants exhibited slightly higher CPP values than male participants, whereas age-related differences were generally small; nonetheless, a tendency towards lower CPP values was observed with increasing age, particularly in the oldest participant group. This finding is consistent with previous reports describing age-related changes in phonatory stability associated with presbyphonia (1).

From a voice therapy perspective, higher CPP values generally reflect a more periodic voice signal with a more prominent harmonic structure and are therefore considered indicative of more stable phonation. The comparatively high CPP values observed during Fröschels' chewing method, Velum Exercise 1, and—relative to the remaining exercises—Fröschels' impact exercises therefore suggest that these exercises elicited relatively stable phonation in healthy speakers. For Fröschels' impact exercises in particular, the comparatively high CPP values may indicate that these tasks promote more complete vocal fold closure and more stable vocal fold oscillation, resulting in a clearer harmonic structure (1). In contrast, diaphragm training and Velum Exercise 3 consistently produced the lowest CPP values. However, this finding should not be interpreted as reduced vocal performance: these exercises primarily serve preparatory or functional therapeutic purposes, and their primary objective is not to maximize acoustic voice quality during task execution but rather to facilitate breathing, respiratory support, coordination, resonance, or muscle activation. Consequently, comparatively lower CPP values in these exercises are likely to reflect the specific phonatory demands of the respective task rather than diminished vocal function.

The increased variability observed for Velum Exercise 2, together with the consistently higher median CPP values in female participants across all age groups, indicates greater interindividual variation in the acoustic characteristics of this exercise. In contrast, diaphragm training exhibited the smallest interquartile ranges despite its comparatively low CPP values, indicating very limited interindividual variability in CPP among healthy participants. While the acoustic characteristics of diaphragm training differed from those of the more strongly phonated exercises, the narrow distribution suggests that healthy speakers produced relatively homogeneous cepstral patterns during this task.

Several methodological factors may explain these comparatively low CPP values. First, recordings were acquired using smartphone microphones under semi-controlled, ecologically valid conditions rather than calibrated laboratory environments. Device-specific signal processing, including automatic gain control, noise reduction, and other proprietary algorithms, may systematically influence cepstral measures. Second, CPP was intentionally calculated from complete speech recordings without removing speech pauses. This approach was chosen to reflect the conditions required for future quasi-real-time acoustic feedback within the LAOLA application but may also contribute to lower overall CPP values.

The close agreement between the complete dataset and the open-access subset demonstrates that the reduced dataset preserves the overall CPP distributions across exercises, sexes, and age groups. Although slightly greater dispersion was observed among younger male participants in selected exercises, the overall distribution patterns remained highly comparable, supporting the representativeness of the open-access subset for future methodological research and algorithm development.

Finally, the observed exercise-specific differences underline that CPP reference values should always be interpreted in relation to the underlying therapeutic task. Because the analyzed exercises differ substantially with respect to respiratory activation, articulatory demands, resonance, and phonatory behavior, a single universal CPP reference value would not adequately reflect physiological voice production across all exercises. Instead, exercise-specific reference values obtained under comparable recording conditions appear to provide a more appropriate basis for future acoustic monitoring within smartphone-based voice therapy applications such as LAOLA. Whether these exercise-specific CPP patterns can reliably distinguish healthy from dysphonic speakers under identical recording conditions should be investigated in future studies involving individuals with voice disorders.

In comparison to normative data published for instance by Murton et al. (18), CPP values were consistently lower than should be expected for persons without any speech pathology. According to Murton et al. (18), a CPP of 9 dB should be regarded as a cutoff, with values below indicating dysphonia (18). Since median values for all exercises were below 8 dB for our healthy participants, the normative cutoff value cannot be applied to our data, nor is it likely to be applicable to future recordings with the LAOLA app, given that comparable recording conditions (smartphone-based, real-time capable) are planned for its clinical use. We propose two possible explanations for these low CPP values. Firstly, since our recordings were made with smartphones in rooms that were not specifically soundproofed, and since we do not know how the mobile device processes the signal during recording (noise cancellation, automatic gain control, etc.), the recordings were likely systematically altered in comparison to calibrated and standardized voice recordings, which could explain the low CPPs. Secondly, although calculating the CPP without removing speech pauses was a deliberate decision with a view toward a possible future quasi-real-time calculation of the CPP as feedback for users, it could also explain the comparatively low CPP values. Since real-time analysis and smartphone recording in normal living and working environments will be the conditions under which a future app must function, these lower CPP values must, for the time being, be regarded as an inherent characteristic of the intended recording conditions. A future calculation of CPPs for people with voice disorders under the same recording conditions should show whether a difference between people with and without dysphonia can still be detected, and whether we would then simply need to use appropriately adjusted cutoff values when providing feedback to users.

A further, and important, factor likely contributing to the comparatively low CPP values observed here is the well-documented task dependency of CPP reference values. Normative CPP cutoffs are not universal but vary considerably depending on the type of speech material from which they were derived, with sustained vowels consistently yielding higher cutoff values than connected or running speech (26). In their investigation of cepstral-spectral acoustic analysis for classifying dysphonia severity, İncebay et al. (26) demonstrated that the discriminative power of CPP differed markedly between the sustained vowel and connected speech contexts, underlining that CPP-based severity classification cannot be generalized across speech tasks (26). This pattern is also evident within the normative data of Murton et al. (18): using Praat, they reported a CPP cutoff of 14.45 dB for sustained/a/ vowels compared to only 9.33 dB for connected speech (Rainbow Passage reading), and using ADSV, 11.46 dB vs. 6.11 dB, respectively. It therefore appears likely that the 9 dB cutoff used here for comparison originates from connected, read speech rather than sustained vowel production, and even this connected-speech cutoff was derived from a standardized reading passage that differs considerably from the short, structured, and partly non-verbal phonation tasks employed in the present voice therapy exercises (e.g., sustained sounds, syllable repetitions, or clinically instructed articulation patterns). This task-dependent nature of CPP reference values constitutes an important, additional explanation for the comparatively low CPP values observed across all exercises in this study and further reinforces that normative cutoff values derived from other speech tasks cannot be directly applied to the present dataset. This limits the clinical transferability of the reported CPP values and should be taken into account when interpreting the dataset for diagnostic or monitoring purposes.

Ambient noise might also have influenced the CPP results, rather than merely representing a limitation on generalizability. Ensuring a minimum signal-to-noise ratio is also technically feasible for home use, as planned for LAOLA: in the future, the application could measure the noise floor prior to recording and prevent recording from starting below a defined threshold. Participants could be given explicit instructions to this effect.

As the analysis of the cross-correlation was carried out with three age groups and two sexes, the following hypotheses could explain the scatter and mean values:

- Older groups might show greater deviations in *diaphragm exercises* or *Fröschels' impact exercises* due to losses in their strength, breath control, and articulation.
- Exercises with semantic complexity [e.g., *Fröschels' chewing method (words)*] are strongly influenced by individual speech patterns—this increases the variance.

The cross-correlation analysis was intended to assess the temporal standardization of exercise execution rather than acoustic similarity between voices. *Fröschels' chewing method (sounds and syllables)* and *Velum exercise 1* in particular show high correlation and low variance while *diaphragm exercises* and *Fröschels' chewing method (words)* exhibited greater variability. This is consistent with the higher number of outliers observed for these exercises, where participants did not perform the exercise as intended. Together with the participant feedback, this may indicate greater uncertainty during the execution of these exercises. Combining cross-correlation, participant feedback, and outlier analysis suggests that objective measures may provide useful insights into the perceived difficulty of individual exercises in a standardized setting. A combination of easier and more challenging exercises may therefore be beneficial for the LAOLA exercise repertoire.

This connection could be examined more explicitly in future work. By binarizing the audio signals, the cross-correlation analysis focuses on temporal speech activity rather than acoustic characteristics such as intensity or spectral content. Consequently, the results primarily reflect the degree to which participants performed the exercises in a temporally standardized manner. While this approach does not allow conclusions about simultaneous acoustic changes, temporal standardization may represent an important prerequisite for future AI-based analyses, as more consistent exercise execution can facilitate the development and validation of automated assessment methods.

Given the specificity of the dataset and the structured nature of the voice tasks, future analyses should prioritize intraindividual monitoring over comparisons with generic normative data. The establishment of exercise-specific reference ranges could facilitate more individualized and clinically relevant feedback. The study was informed by the “Checklist for Instrumental Acoustic Voice Quality Assessment” proposed by Brockmann-Bauser and de Paula Soares (15). In the Appendix (Table B), we summarize the points from their checklist that are relevant to us. This approach contributes to ongoing efforts to improve the transparency, reliability, and clinical applicability of acoustic voice monitoring in speech-language therapy. Moreover, our open access data contribute to understanding cross-linguistic and language-specific aspects of voice acoustics (15). These data may serve as a valuable resource for the broader scientific community working on dysphonia.

## 5 Conclusion and outlook

We have introduced and evaluated the standardized speech dataset “LAOLA—voice therapy recording audio dataset 1”. It consists of 175 structured recordings of voice exercises relevant to dysphonia therapy. Of the test subjects, 74% consented to open access. The recording setup was generally well accepted among participants and is suitable for data collection. However, specific aspects were also identified that could further improve future recordings, such as adequate practice time and clearer instructions.

Our results indicate that the standardized and analyzable voice recordings are feasible under a consistent technical setup and everyday conditions using smartphones. The latter refers to using a smartphone for voice training at a time of one's own choosing, without needing to be in a dedicated training room.

Three findings are central in this respect. First, mean pitch values fell within published normative ranges and showed the expected sex- and age-related differences, whereas intensity and CPP values were systematically lower than laboratory-derived reference values, reflecting task-specific vocal demands and uncalibrated smartphone recording.

Second, CPP, pitch and intensity did not differ between the recording rooms, indicating that the dataset is robust under varying but acoustically comparable conditions.

Third, the cross-correlation analysis revealed a consistent temporal structure of exercise execution across participants, with onset differences of less than one second.

Taken together, the acoustic analyses demonstrate that the dataset can be interpreted meaningfully under these recording conditions, while underscoring that task-specific and technical influences must be considered when comparing the data with generic normative values. Accordingly, the results support the use of exercise-specific and intra-individual reference frameworks for future analyses of therapeutic voice performance. In future work, ambient noise, room acoustics, recording distance, and device-related variability should be analyzed in greater detail to enable more precise feedback.

Beyond the present analyses, the high degree of standardization of the dataset may provide a useful basis for future machine-learning applications, as structured and temporally aligned recordings facilitate targeted analysis and model development. In this way, the dataset may support future automated analyses as well as the development of digital tools that complement established approaches in speech-language pathology. Future studies should extend the present approach to participants with diagnosed dysphonia in order to evaluate clinical applicability under real-world conditions. Within the broader perspective of the LAOLA project, this includes further work on speech signal processing under everyday acoustic conditions and the possible development of additional open datasets incorporating both vocal and movement-related information. Ultimately, such work may contribute to more individualized and patient-centered voice therapy, including home-based training supported by objective biofeedback.

## Data Availability

The datasets presented in this study can be found in online repositories. The can be found here: https://zenodo.org/records/22077896.

## Footnotes

^1^“LAOLA” is a proper name based on OLA (“Oldenburger Logopädie App”) and not an acronym.

^2^The participants have given their written consent for their audio recordings to be published online. The blank form for this can be found in the appendix.

^3^This checklist can, for example, be viewed in Supplementary A

^4^URL: https://www.drks.de/search/de/trial/DRKS00033225

^5^Content: place the feet flat and parallel on the floor, tilt the pelvis slightly forward, pull the sternum up, pull the shoulders slightly back, pull the chin slightly towards the chest, stretch the neck upwards

^6^This was not reflected in vocal parameters, as one person was dizzy and another was experiencing arm pain due to a recent surgery.

## Supplementary material

Supplementary A The TIDieR (Template for Intervention Description and Replication) Checklist: Information to include when describing an intervention and the location of the information.

## Appendix

**Table A:** Overview of the distribution of subjects according to Federal Employment Agency (KldB) 2018 (27; *own presentation).*

Classification of professions	Number of subjects	Percentage (%)
1. Agriculture, forestry, animal husbandry, and horticulture	0	0
2. Raw material extraction, production, and manufacturing	4	3
3. Construction, Architecture, Surveying, and Building Technology	3	2
4. Natural Sciences, Geography, and Computer Science	8	6
5. Traffic, logistics, protection, security	2	1
6. Commercial services, trade in goods, distribution, hotel, and tourism	13	10
7. Business Organization, Accounting, Legal, and Administration	5	4
8. Health, Social Affairs, Teaching, Education	28	21
a. *noted separately in this grouping: SLPs*	31	23
9. Linguistics, Literature, Humanities, Economics, Media, Art, Culture, and Design	2	1
10. Military	0	0
11. Additional groupings		
*a. Trainees/Students*	9	7
*b. Pensioners*	13	10
*c. Inactive persons*	5	4
*d. Voice Patients*	12	9
*e. No specification*	*38*	*23*

**Table B:** Checklist for instrumental acoustic voice quality measurements *(own presentation).*

Conditions met for instrumental acoustic voice quality measurements
✓ German is the native language for all participants
✓ Standardized process
✓ Focus on a few relevant parameters [pitch mean, intensity (SPL), CPP]
✓ Presentation of all devices used
✓ Description of the acoustic environment (identical room size)
✓ Presentation of the exercises: same exercises in randomized order, no possibility to repeat the exercises, etc.
✓ Presentation of the hardware, software, recording devices, and distance to the microphone
✓ Reminder of the subjects to speak at normal volume during the recording
✓ Population comparable between sex, three age groups, voice production and use, and further socio-demographic data
✓ Objective and valid measurement of vocal performance
✓ Large data material (400 GB) with open access data
✓ Entire phonation used for the analysis
✓ Understanding the relationship between perceptual and instrumental acoustic results: Analysis based on Praat and manual selection of outliers
✓ Inclusion of feedback from the participants
✓ Comprehensive reporting of the study
