# Supplementary material for: Development and evaluation of a voice dataset for voice therapy exercises: quantitative audio analysis and user feedback from a cross-sectional study
Source: Front Digit Health. 2026 Sep 11;8:1835534. doi: 10.3389/fdgth.2026.1835534 (PMC13614008; doi:10.3389/fdgth.2026.1835534)
Supplement: Supplementary file 1 [file Datasheet1.docx]

**The TIDieR (Template for Intervention Description and Replication) Checklist:**

Information to include when describing an intervention and the location of the information

| **Item number** | **Item** |  |
| --- | --- | --- |
|  |  |  |
|  | **BRIEF NAME** |  |
| **1.** | Provide the name or a phrase that describes the intervention. | ✓ |
|  | **WHY** |  |
| **2.** | Describe any rationale, theory, or goal of the elements essential to the intervention. | ✓ |
|  | **WHAT** |  |
| **3.** | Materials: Describe any physical or informational materials used in the intervention, including those provided to participants or used in intervention delivery or in training of intervention providers. Provide information on where the materials can be accessed (e.g. online appendix, URL). | ✓ |
| **4.** | Procedures: Describe each of the procedures, activities, and/or processes used in the intervention, including any enabling or support activities. | ✓ |
|  | **WHO PROVIDED** |  |
| **5.** | For each category of intervention provider (e.g. psychologist, nursing assistant), describe their expertise, background and any specific training given. | ✓ |
|  | **HOW** |  |
| **6.** | Describe the modes of delivery (e.g. face-to-face or by some other mechanism, such as internet or telephone) of the intervention and whether it was provided individually or in a group. | ✓ |
|  | **WHERE** |  |
| **7.** | Describe the type(s) of location(s) where the intervention occurred, including any necessary infrastructure or relevant features. | ✓ |
|  | **WHEN and HOW MUCH** |  |
| **8.** | Describe the number of times the intervention was delivered and over what period of time including the number of sessions, their schedule, and their duration, intensity or dose. | ✓ |
|  | **TAILORING** |  |
| **9.** | If the intervention was planned to be personalised, titrated or adapted, then describe what, why, when, and how. | ✓ |
|  | **MODIFICATIONS** |  |
| **10.** | If the intervention was modified during the course of the study, describe the changes (what, why, when, and how). | ✓ |
|  | **HOW WELL** |  |
| **11.** | Planned: If intervention adherence or fidelity was assessed, describe how and by whom, and if any strategies were used to maintain or improve fidelity, describe them. | ✓ |
| **12.** | Actual: If intervention adherence or fidelity was assessed, describe the extent to which the intervention was delivered as planned. | ✓ |
